# Oral soft tissue biopsies in Oporto, Portugal: An eight year retrospective analysis

**DOI:** 10.4317/jced.52677

**Published:** 2015-12-01

**Authors:** Manuel-Moreira Guedes, Rui Albuquerque, Marta Monteiro, Carlos-Alberto Lopes, José-Barbas do Amaral, José-Júlio Pacheco, Luís-Silva Monteiro

**Affiliations:** 1 MSc. Instituto Superior de Ciências da Saúde Norte, CESPU, Paredes, Portugal; 2 PhD, MS, DDS. Oral Medicine Department, Birmingham Dental Hospital. School of Dentistry, University of Birmingham. Birmingham B4 6NN, United Kingdom; 3 MD. Internal Medicine Department, Centro Hospitalar do Porto, Hospital de Santo António-Porto, Portuga; 4 PhD, MD. Molecular Pathology and Immunology Department, Instituto de Ciências Biomédicas Abel Salazar (ICBAS), Porto University, Porto, Portugal; 5 PhD, MD. Stomatology Department, Centro Hospitalar do Porto, Hospital de Santo António-Porto, Portugal; 6 PhD, MSc, DDS. Medicine and Oral Surgery Department, Dental Sciences Group – Health Sciences Research Centre, Instituto Superior de Ciências da Saúde Norte, CESPU, Paredes, Portugal

## Abstract

**Background:**

The diseases that affect the oral cavity are wide and diverse, comprising a broad spectrum of either benign or malignant lesions. However, few histological-based studies were performed for the evaluation of oral cavity lesions, and very few directed to oral soft tissue pathology. The aim of this study was to carry out pioneering research, within a Portuguese population, to determine the frequency and characteristics of oral malignancies, potential malignant disorders, and soft benign tissues pathologies submitted for biopsy in a north Portugal (Oporto) hospital population.

**Material and Methods:**

We performed a retrospective study of soft tissue, oral cavity biopsies, in a hospital north of Portugal (Oporto) between 1999 and 2006. We analysed information on gender, age, location of the lesion, and the histopathological diagnosis.

**Results:**

A total of 1042 oral biopsies were observed, 557(53.5%) in females and 485 (46.5%) in males, with a mean age of 51.7 years (S.D. ±17.6). The topographic location most frequently affected was labial mucosa (n=306). Considering the nature of the lesions, 700 (67.2%) corresponded to non-neoplasic lesions, 45 (4.3%) to potentially malignant disorders, and 297 (28.5%) to neoplasms (93 benign and 204 malignant). Non-neoplasic lesions were more prevalent in female gender (59.9%) when compared with potentially malignant disorders (46.7%) and neoplasms (39.4%) (*P*< 0.001). Non-neoplasic lesions presented the lower mean age (49.2±17.6) and potentially malignant disorders the highest mean age (60.5±14.5) (*P*< 0.001). The most common lesion of entire sample was fibro-epithelial hyperplasia (n=186; 17.9%), followed by squamous cell carcinoma (n=158; 15.1%).

**Conclusions:**

Fibro-epithelial hyperplasia, followed by squamous cell carcinoma, was the most common pathologies. This pioneering study provided, for the first time, data about the proportion of squamous cell carcinoma when compared with benign conditions in a Portuguese hospital population.

** Key words:**Oral biopsies, oral cavity, oral pathology, Portugal, soft tissue lesions.

## Introduction

The diseases that affect the oral cavity are wide and diverse, comprising a broad spectrum of either benign or malignant lesions. The diagnosis of many of them through biopsy is an essential part of oral medicine, allowing us to characterize them histopathologically and then grouping them in a more systematic and coherent way.

In the international scientific literature, most available and predominant studies on oral cavity lesions have an epidemiological or clinical nature, focusing on the incidence of specific pathological condition for a given population, for a certain age group, and many times without histological diagnosis (1-9). Few histological-based studies including patients of all ages were performed for the evaluation of oral cavity lesions, and very few directed to oral soft tissue pathology (10-14). To our knowledge, this has never been carried out in a Portuguese population. With the rise of oral cancer in Portugal (15) and with the recent learning programmes, to promote early recognition of oral cancer, as well as general dental practitioners to carry out biopsies in dental practice environments, we believe it is important to obtain baseline data for future comparisons to understand the impact of this. Consequently, the aim of this study is to carry out a pioneering study in a Portuguese North population to determine the frequency of oral malignancies and potential malignant disorders in the soft tissues compared with the frequency of other soft benign tissue lesions of oral cavity submitted to biopsy in a hospital population, north of Portugal (Oporto).

## Material and Methods

A total of 1839 histological diagnoses of oral biopsies (ICD 10: C00-06) were collected from the Pathology Department of the Hospital de Santo António, Oporto - Portugal (HSA), over an 8-year period (1999-2006). The study was approved and performed according to the institutional review board of the hospital. All histopathological reports and correspondent clinical information on the exam were analysed and entered into a database, including information on gender, age, location of the lesion, clinical diagnosis and the histopathological description and diagnosis. For patients with more than one biopsy, we included only the definitive diagnosis (or more representative), in order not to overstate the sample. Repeated biopsies of already diagnosed lesions were excluded. Cases with unclear or missing data, or inconclusive pathological results were excluded. Bone lesions were excluded. From the total amount of the obtained diagnoses -1839, 797 of them were excluded, leading to a final sample of 1042 cases for analysis. All cases were reviewed and reclassified by an experienced pathologist to confirm the initial diagnosis. The diseases were classified according to a classification adapted from the one proposed by ICD-DA (International Classification of Diseases to Dentistry and Stomatology) and by the WHO classification of tumours (2005). For analysis proposes the diagnosis were divided into three groups including non-neoplasic lesions, potentially malignant disorders, and neoplasic lesions and also subdivided into 10 major subcategories: normal tissue, inflammatory / infectious lesions, cystic lesions, adaptive reactions, potentially malignant disorders, autoimmune / metabolic diseases, vascular / hemodynamic anomalies, hamartomatous lesions and congenital alterations, benign neoplasms and malignant neoplasms.

The collected data were inserted into a database created in the IBM SPSS Statistics version 21.0 software (IBM Corporation, NY, US). The results were presented in absolute and relative frequency. We used *Anova* test to analyze the continuous variables and chi-square test for categorical variables, considering differences statistically significant at *P* < 0.05.

## Results

**Figure 1:**
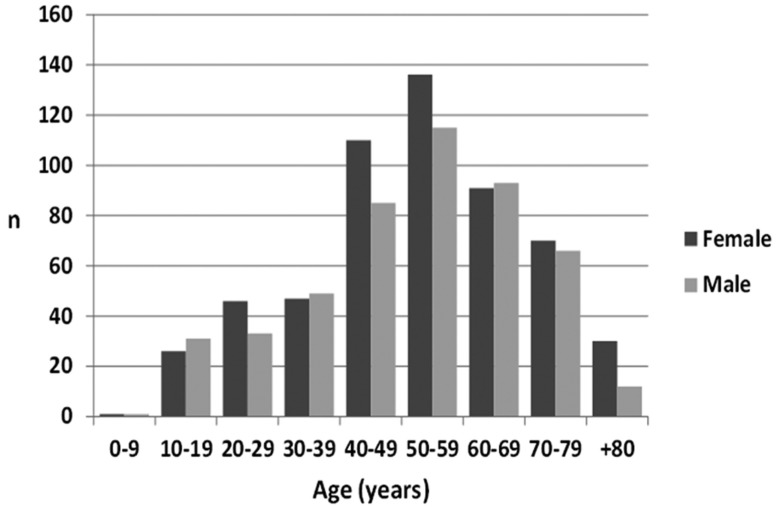
Distribution of the cases by gender and age.

From the 8 year period, 1042 oral biopsies were included in this analysis, 557 (53.5%) females and 485 (46.5%) males, with a female:male ratio of 1.1. Regarding age, patients were between 3 and 100 years-old, being on average - 51.7 years (S.D. ±17.6) and the 6th the most affected decade (Fig. 1).

**Figure 2:**
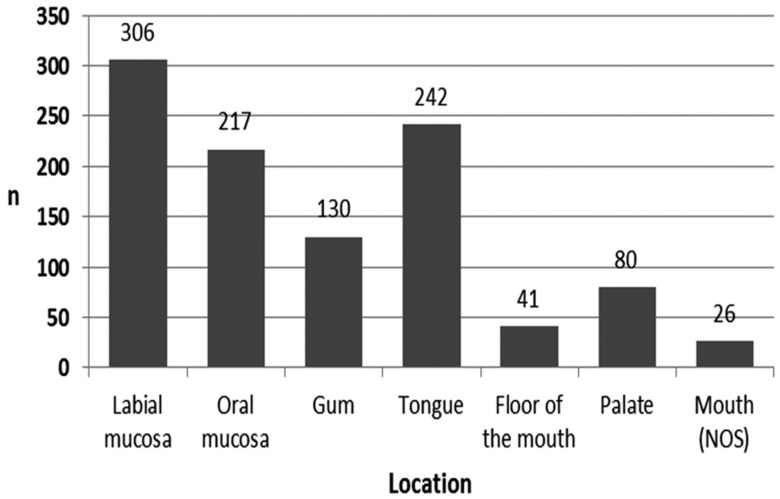
Distribution of the cases by topographic location (NOS = no-otherwise specified).

The topographic location most frequently affected was labial mucosa (n=306), followed by the tongue (n=242), oral mucosa (n=217), gums (n=130), palate (n=80), floor of the mouth (n=41), and mouth no-otherwise specified (NOS) (n=26) (Fig. 2).

Considering the nature of the obtained diagnosis, 700 (67.2%) lesions corresponded to non-neoplasic diagnosis, 45 (4.3%) were potentially malignant disorders, and 297 (28.5%) to neoplasms. From the 700 non-neoplasic diagnosis, the most common subcategory was the adaptive / reactive lesions (n=295), followed by inflammatory / infectious lesions (n=161), cystic lesions (n=71), autoimmune or metabolic conditions (n=67), vascular or hemodynamic anomalies (n=51), and by hamartomatous or congenital anomalies (n=15). There were 40 diagnoses with tissue without pathological alterations (normal tissue). From the 297 neoplasms, 93 were benign and 204 were malignant.  Table 1 sum up the number of diagnostic groups and subcategories by gender, mean age, and predominant topographic location.

**Table 1:**
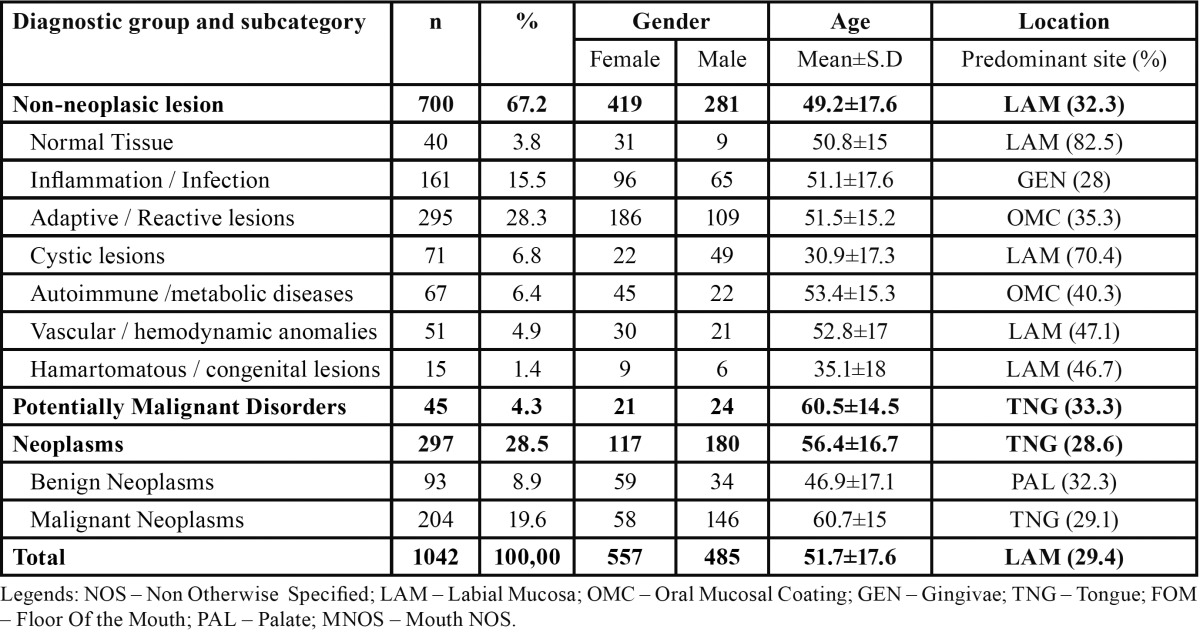
Number of diagnoses of all groups and subcategories distributed by gender, age and predominant topographic location.

Female gender was more prevalent in non-neoplasic lesions (59.9%) when compared with potentially malignant disorders (46.7%) and neoplasms (39.4%) (*P* < 0.001, *chi-square test*). Non-neoplasic lesions presented the lower mean age (49.2±17.6) and potentially malignant disorders the highest mean age (60.5±14.5) (*P* < 0.001, *anova test*).

 Table 2,  Table 2 (continue) shows the distribution of all non-neoplasic diagnosis by gender, mean age, and predominant topographic location. The most common adaptive / reactive lesion corresponded to fibroepithelial hyperplasia (n=186), more observed in females (n=127), with a mean age of 50.5±15.8 years, and affecting most frequently the oral mucosa (42.5%). The inflammatory / infectious lesions with more cases was the non-specific ulcer (n=39) more common in tongue (38.5%), in females (n=23), and with a mean age of 54.2±18.4. Mucocele (n=63) was the most predominant cystic lesion occurring predominantly in labial mucosa (76.2%), in males (n= 43), and with a mean age presentation of 32.1±17.5 years. On the autoimmune lesions, lichen planus / liquenoid reactions (n=34) were the most prevalent, observed most often in females (n=19), in oral mucosa (75%) with a mean age of 52.8±15.6. The hamartamous / congenital alterations were most represented by melanocytic nevus (n=6) diagnosed most in labial mucosa (66.7%), in males (n=4), and with a mean age of 39.3±18 years. The vascular / hemodynamic anomalies category were most represented by vascular anomalies with 48 cases, most on females (n=27), and in labial mucosa (43.8%), with a mean age presentation of 52.3±17.4 years.

**Table 2:**
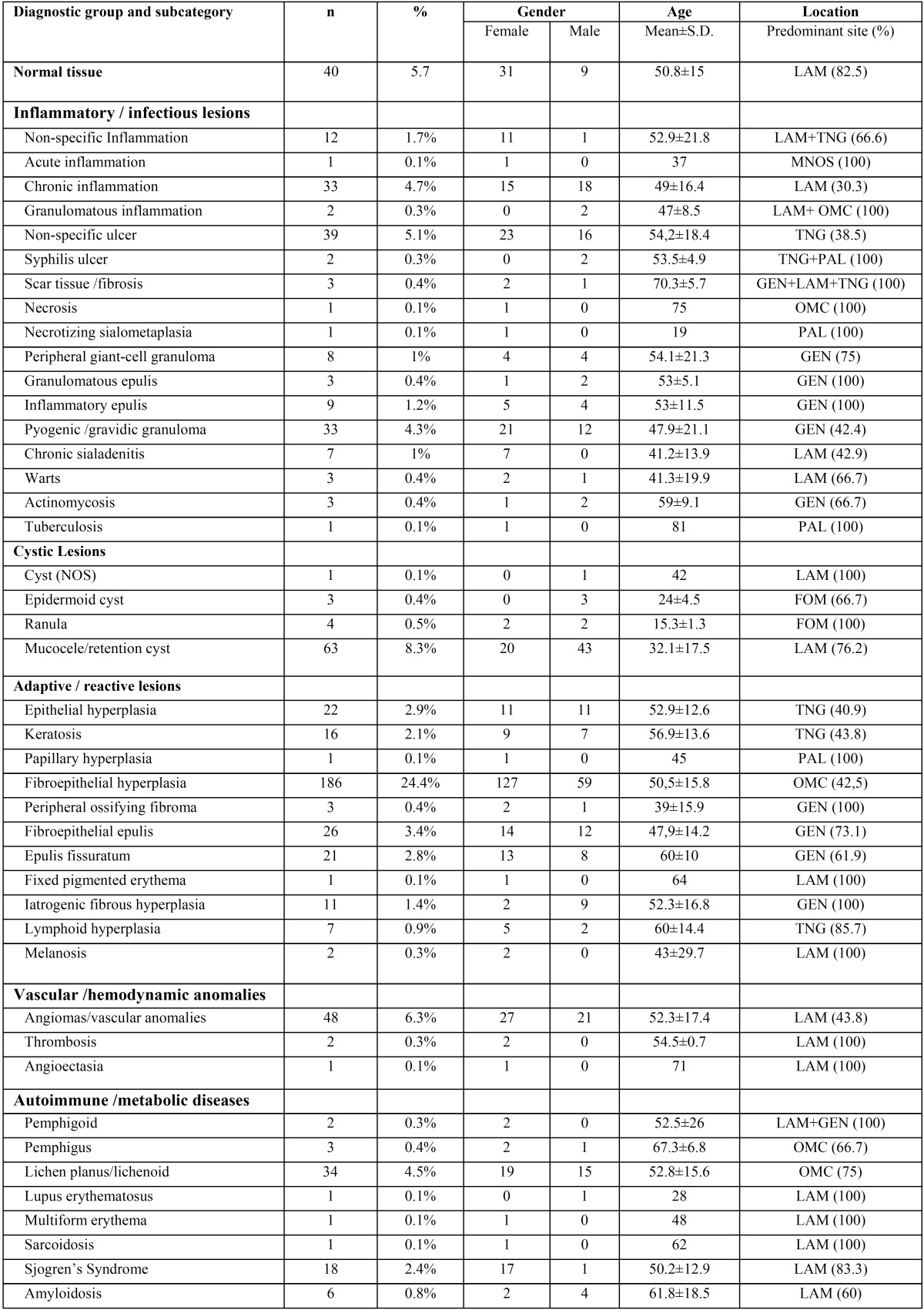
Number of non-neoplasic diagnosis distributed by gender, age and predominant topographic location.

**Table 2 continue:**
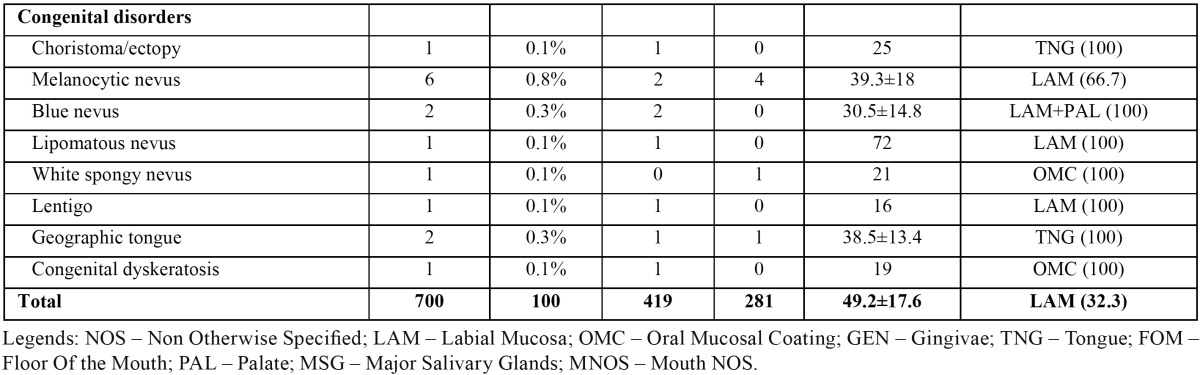
Number of non-neoplasic diagnosis distributed by gender, age and predominant topographic location.

 Table 3 shows the distribution of potentially malignant disorders by gender, mean age, and predominant topographic location. On the potentially malignant disorders, leukoplakia (n=38) was the most prevalent lesion occurring more frequently in males (n=21), in the tongue (37.8%), and with a mean age presentation of 58.8±15.1 years.

**Table 3:**
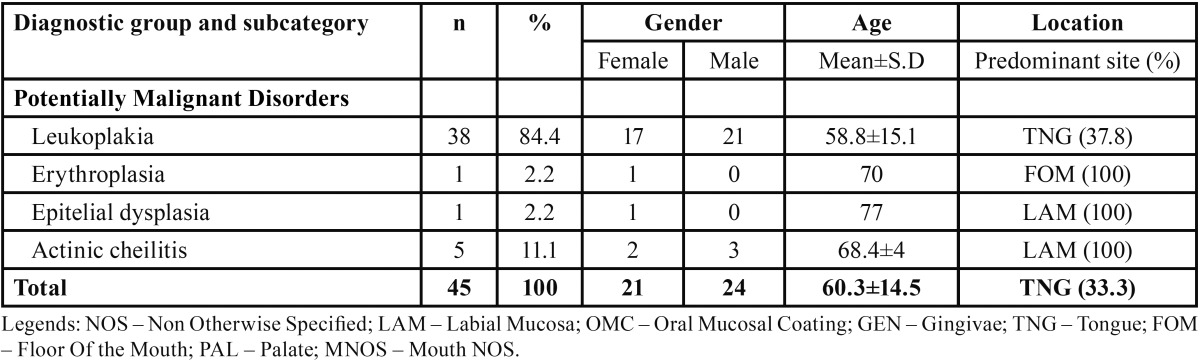
Number of potentially malignant disorders distributed by gender, age and predominant topographic location.

Neoplasic lesions are shown in the  Table 4 distributed by gender, mean age, and predominant topographic location. The most prevalent neoplasm was squamous cell carcinoma (SCC) (n=158), occurring most frequently in males (n=114), with a mean age presentation of 62.4±13.8 years. The most common location affected by SCC was the tongue (32.9%).

**Table 4:**
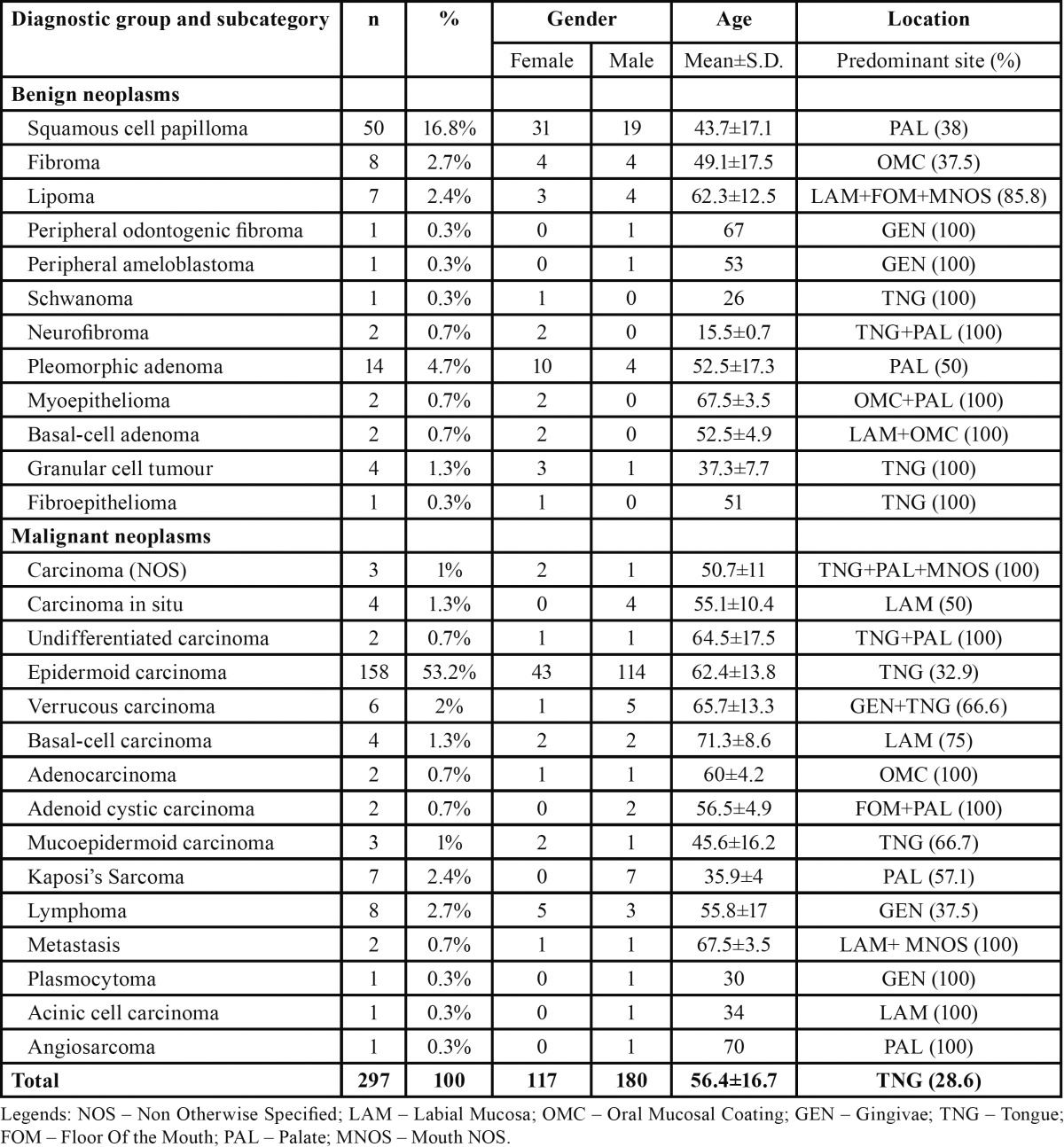
Number of potentially malignant disorders distributed by gender, age and predominant topographic location.

**Figure 3:**
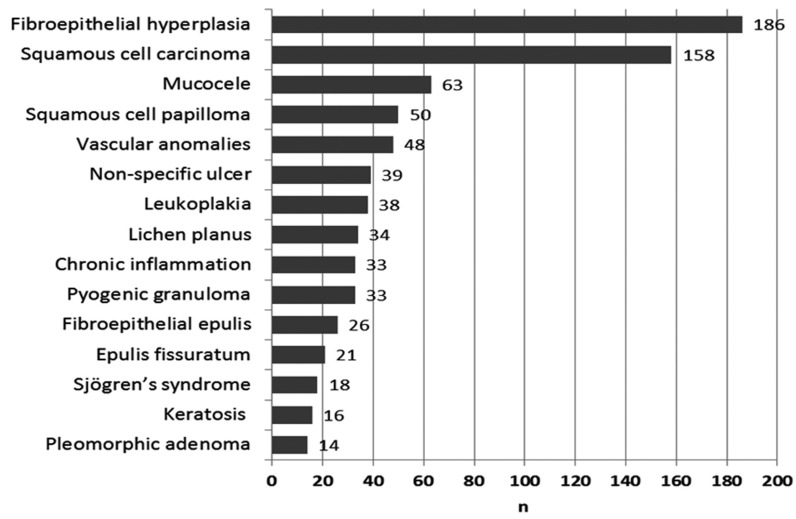
The 15 most common histological diagnoses (1999-2006).

The 15 most common diagnoses are shown in figure 3. These include 777 cases, about 75% of the whole obtained specimens. If we consider the most common diagnoses divided by age presentation in a young group (0 to 24 years), adult group (25 to 59 years) and elderly group (+ 60 years) we observed that mucoceles (n=27; 29.3%) were the predominant lesions in the young group, the fibroepithelial hyperplasia were the most common in the adult group (n=137; 19.9%), and SCC (n=71; 27%) in the elderly group. Considering the most prevalent lesions by gender, fibroepithelial hyperplasia (n=127; 22.8%) was the most common lesion in females, and SCC (n=115; 23.7%) in males.

## Discussion

This first data collected in the North Portuguese patients will provide relevant information for further studies, especially now that new programmes are being introduced in Portugal to promote early detection of oral cancer. In view of this, we performed a descriptive and retrospective analysis of a large group of soft tissue oral lesions, submitted to biopsy with a definitive histological report in a hospital north of Portugal. The interpretation of the results must be made while keeping in mind the limitations of this type of retrospective study. Authors acknowledge the limitations of the division into 3 groups and consequently 10 subgroups in which some of the conditions overlap. Although the high number of cases selected from the main and central hospital of the city, they maybe not representative of the entire Oporto population. We only evaluated lesions that were submitted to biopsy, which means that there were some oral lesions (without biopsy indication) that were not included here such as recurrent afthous stomatitis, or some forms of gingivitis. Nevertheless, the presence of the histological description augments the accuracy of diagnosis, when compared with studies that have only a clinical diagnosis. Moreover, we described the predominant topographic location for each lesion which we believe to be an important contribution to a better understanding of the epidemiology of injuries when analyzed individually.

The data obtained in the present sample shows a slight tendency for oral soft tissue diseases to occur more in females. This trend is also reported in literature by others (8,9,11). These results could be explained by the fact that women tend to seek healthcare providers more often, as well as there being a higher prevalence of women in the population of north Portugal (according to Census of 2011). This apparent female prevalence was, however, subverted when we consider the particular group of neoplasms especially the malignant neoplasms, similar to the published data on the epidemiology of oral lesions (12,16).

All ages can be affected by oral disease or oral lesions and this was observed in our sample with the minimum age of 3 years and maximum of 100 years old. However, most of the cases were found in the 4th, 5th and 6th decades of life. This age of presentation was related with the type of oral lesion where non-neoplasic lesions were found in early decades and potentially malignant disorders and cancers were found in advanced decades. It is well stablished that oral potentially malignant disorders and oral cancer are more seen in the elderly (8,17-20).

The fibroepithelial hyperplasia (n=186) was the most common diagnosis of the entire sample (17.9%), affecting more often females, around the 5th decade of life and being predominantly located in the buccal mucosa. The majority of the published literature confirms the obtained data (7,9,10,21,22). In fact, the adaptive / reactive lesions were the most pathological group found in our study. This shows that the adaptive capacity of the oral mucosal tissues can modify their morphology and function to face several traumatic and chronic pathological stimuli, such as maladapted prosthesis or malocclusion. We must bear in mind that true beneficial treatment of these lesions is not only the elimination of the lesion but essentially the elimination of the cause of the lesions.

Ulcers (non-specific and syphilitic) (n=41) were the most frequently diagnosed in the inflammation/infection group, which constituted about 3.9% of the whole observed pathologies, a similar value was observed by Jones and Franklin (7). Other prevalent inflammatory lesions included chronic inflammation and pyogenic/gravid granuloma. The pyogenic granuloma affected most commonly females and revealed a predilection for the gingiva, although in a more discreet percentage of what was reported by Krishnapillai *et al.* (23).

Mucoceles (n=63) were the most common cystic lesion, representing approximately 6% of the whole found pathologies, similar to those reported by Weir *et al.* (16). Males were predominantly affected, results were in line with those observed by Jones and Franklin (7). The low average age of patients (32.1 years) and the labial mucosa location predominance were also concordant with those found by other authors. Chi *et al.* (24) in its series of 1834 mucoceles (81.9%) found that 81.9% of mucoceles in labial mucosa had a mean age of 25 years old. In fact, in a retrospective survey by Wang *et al.* (25) of biopsied oral lesions in paediatric patients the most common lesion were mucoceles which is in accordance with our results, if we only consider the group of young patients (0-24 year-old).

The most autoimmune lesion found in our study was lichen planus (n=34), a result that shows the prevalence of these diseases in oral cavity (7,8,11). There was prevalence in females, and a mean age in the 5th decade of life, similar to that described by Jones and Franklin (7). The second most frequent pathology in this group was the diagnosis of Sjogren’s Syndrome (n=18) having a higher prevalence in females, in accordance with what is reported in the literature (21,26).

The presence of vascular lesions consisted essentially in vascular blood anomalies. We decided to join the “haemangiomas”, “lymphangiomas”, vascular malformations, arteriovenous malformations and varix, in the group of vascular anomalies as the terminology reported in the exams was not systematized. Only infantile hemangiomas or vascular lesions with true neoplasic nature were classified as vascular tumors. Indeed most of the haemangiomas in adults are nowadays considered hamartomatous lesions and are called vascular anomalies (27).

The most representative pathological entity of benign neoplams was squamous papilloma (n=50) followed by pleomorphic adenoma (n=14), which is in accordance with the reports by Al-Khateeb (21), Ali and Sundaram (13) and by Jones and Franklin (7).

The group of potentially malignant disorders constitutes about 4.3% of the total sample, represented more often by leukoplakia (n=38). That constituted 3.6% of all lesions diagnosed, a percentage inside the reported by several studies (7,11). Axell (4) in a survey on oral mucosal lesions in Sweden observed 3.6% true oral leukoplakias. It is possible that this value in our study could be higher as some leukoplakias could be under the epithelial hyperplasia or hyperqueratosis diagnosis.

The rate of malignancies in the present study (19.5%) is superior to those reported by some studies (7,9,11) and closer to the 15% reported by Ali and Sundaram (13) who used 858 biopsied oral soft tissue lesions in Kuwait. Squamous cell carcinoma was the most common malignancy as in the vast majority of reviewed studies (9,11,13,16,22). This reflects not only the prevalence of oral squamous carcinoma but also the importance of biopsy for the diagnosis of this disease. There was a predilection of male patients, with a mean age around 60 year-old, and the tongue was the predominant topographic location (32.9%), which is in agreement with several epidemiologic and clinic-pathological series reported (11-13,19,20,28). Oral cancer is a health problem causing concern in Portugal. In the last decade an increasing trend for oral cancer in the Portuguese population has been reported (15), contributing to ranking Portugal as the second country in Europe with the highest incidence of lip and oral cavity cancer (Globocan, 2012).

In conclusion, the extent of histopathologic soft tissue lesions, affecting the oral cavity of a hospital population for a period of 8 years (1999-2006)has been indeed significant, with almost 90 different histological obtained diagnoses. The most common soft tissue lesions that affect the oral cavity were non-neoplasic pathology, being fibroepithelial hyperplasia, the histological diagnosis more frequent in the entire studied sample. SCC were the second more common diagnosis, found more often in males and in the 6th decade of life. Our study confirms the prevalence and the importance of histopathology in the diagnosis of SCC providing for the first time data about the proportion of squamous cell carcinoma when compared with benign conditions in a Portuguese hospital population. This data can be used for future research to understand the impact of the new learning programmes to promote early diagnosis in oral cancer. We believe that the data on soft tissue of oral cavity presented here may be useful in formulating diagnostic impressions that will be of particular interest to pathologists, oral/maxillofacial surgeons and general dental practitioners.
